# Supplementary figures and images for: Carpal tunnel syndrome and occupational co-exposure to biomechanical factors and neurotoxic chemicals using job-exposure matrices and self-reported exposure: Findings from the Constances cohort
Source: PLoS One. 2025 Sep 15;20(9):e0329324. doi: 10.1371/journal.pone.0329324 (PMC12435683; doi:10.1371/journal.pone.0329324)

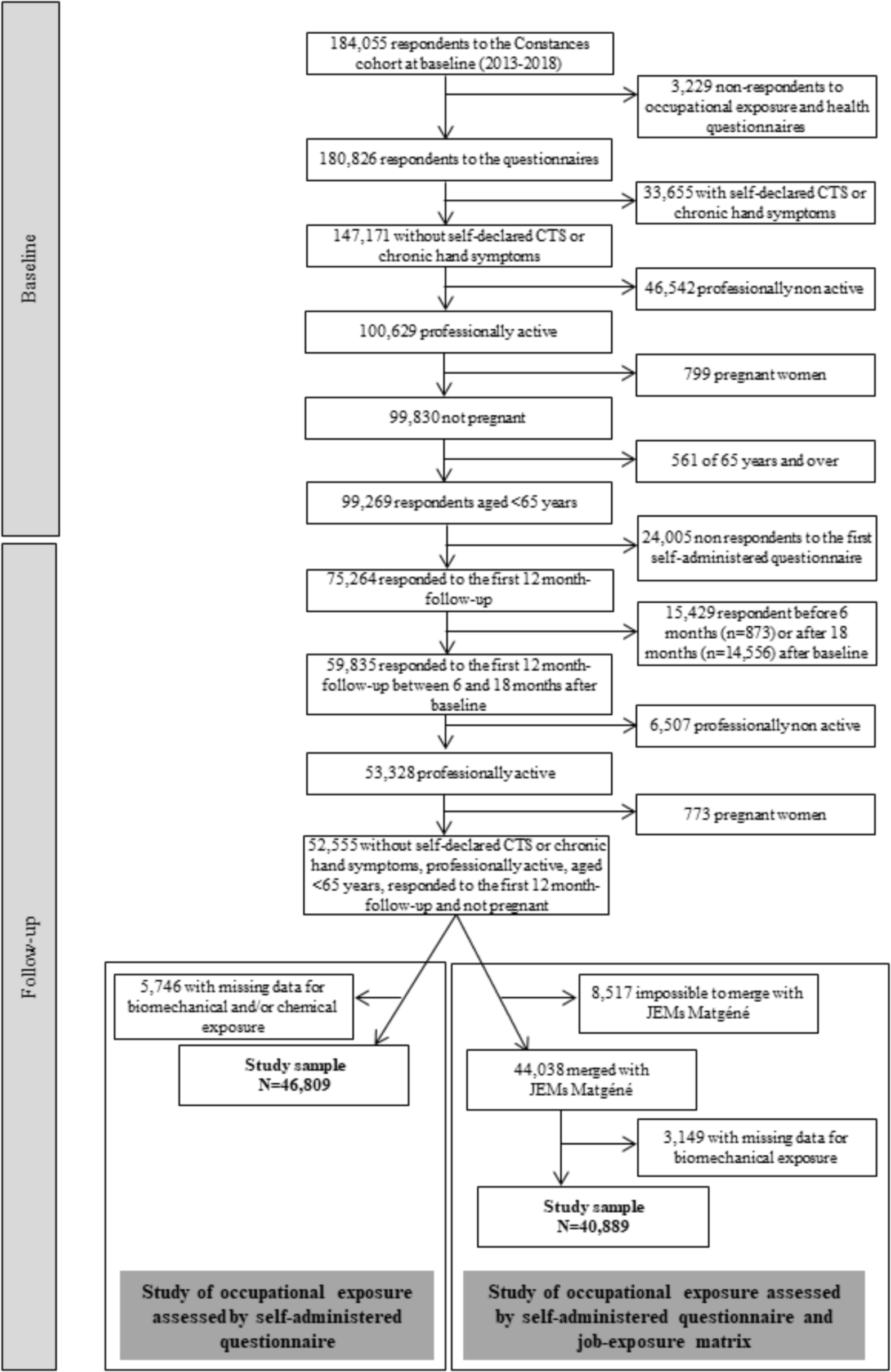

Supplement: S1 Fig — (TIF) [file pone.0329324.s001.tif]

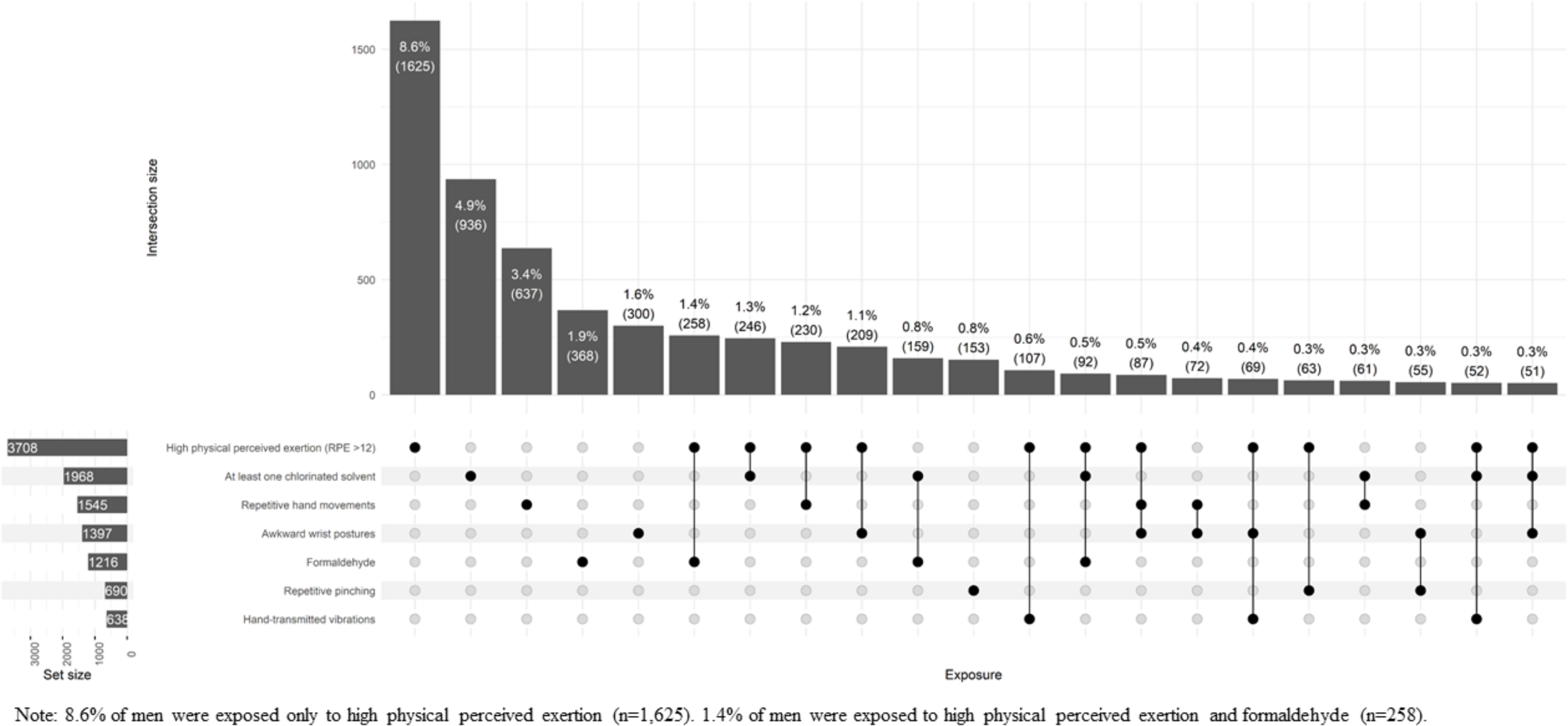

Supplement: S2 Fig — Note: 8.6% of men were exposed only to high physical perceived exertion (n = 1,625). 1.4% of men were exposed to high physical perceived exertion and formaldehyde (n = 258). (TIF) [file pone.0329324.s002.tif]

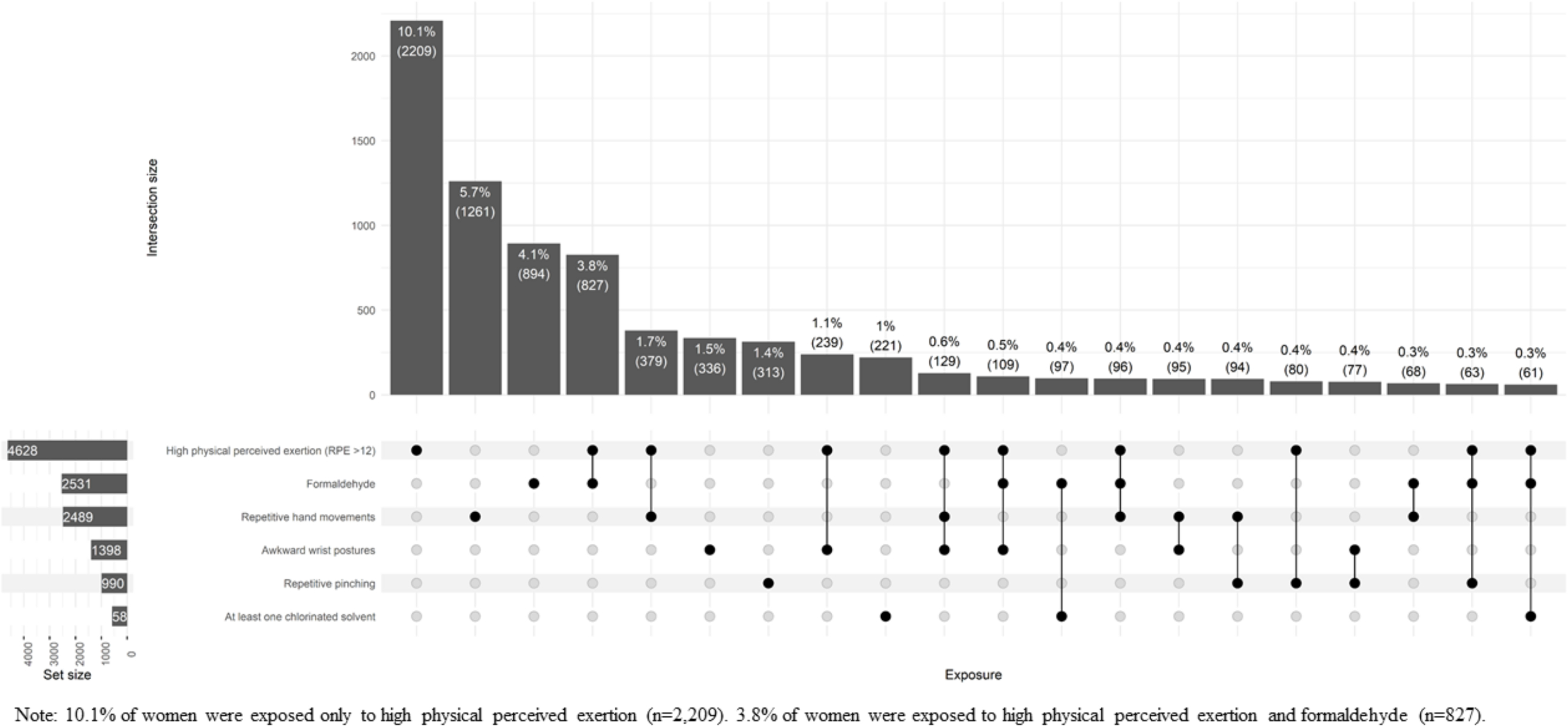

Supplement: S3 Fig — Note: 10.1% of women were exposed only to high physical perceived exertion (n = 2,209). 3.8% of women were exposed to high physical perceived exertion and formaldehyde (n = 827). (TIF) [file pone.0329324.s003.tif]

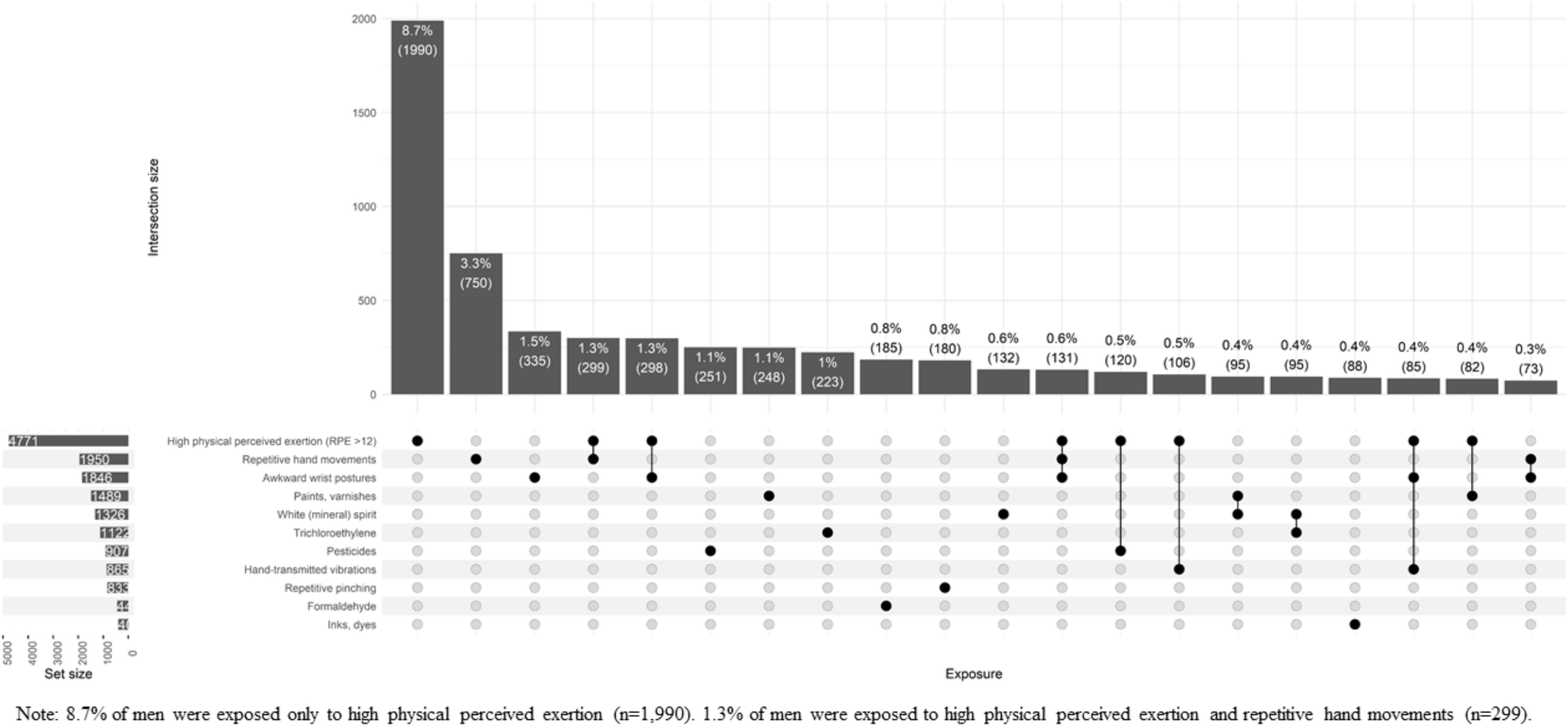

Supplement: S5 Fig — Note: 8.7% of men were exposed only to high physical perceived exertion (n = 1,990). 1.3% of men were exposed to high physical perceived exertion and repetitive hand movements (n = 299). (TIF) [file pone.0329324.s005.tif]

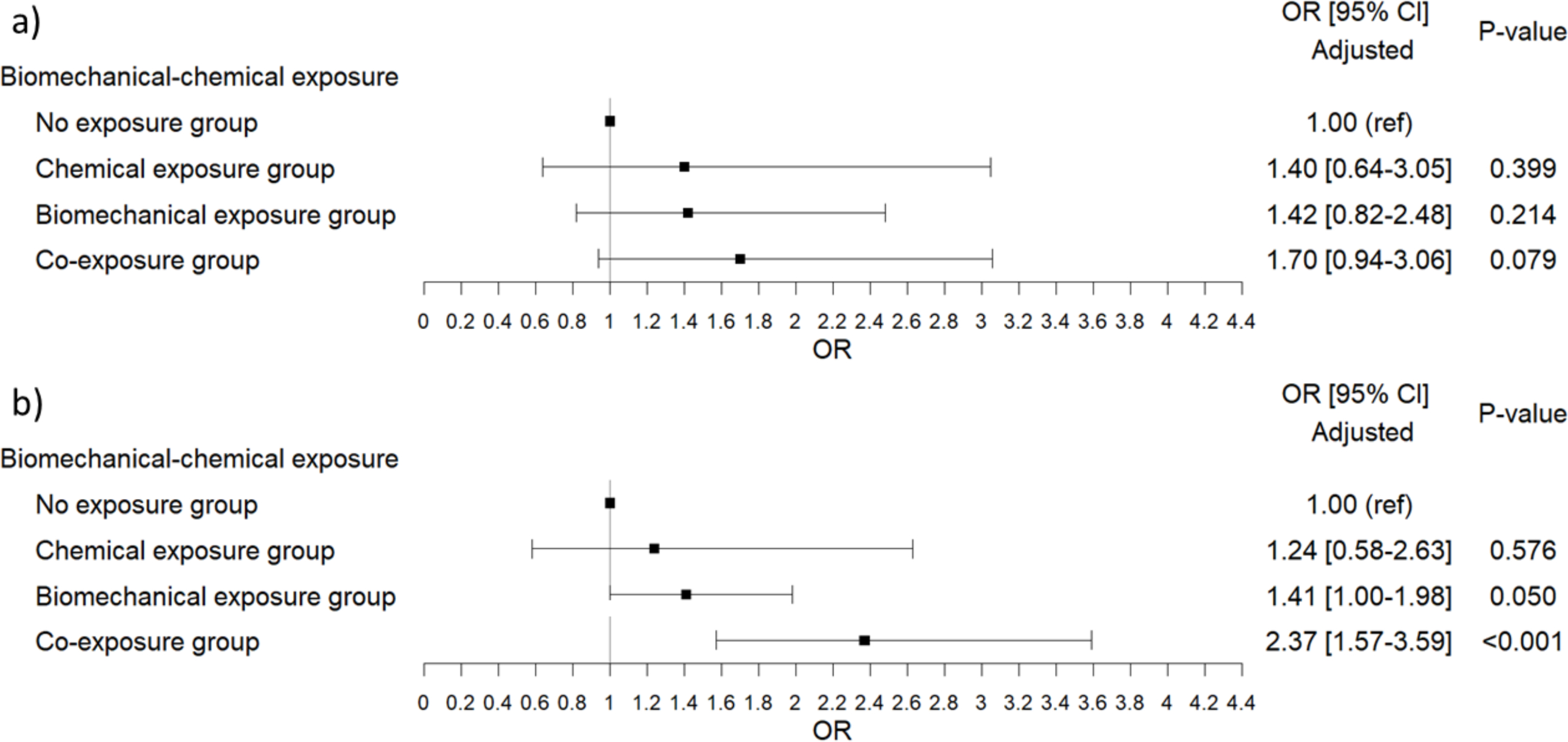

Supplement: S6 Fig — Note: 13.0% of women were exposed only to high physical perceived exertion (n = 3,121). 2.2% of women were exposed to high physical perceived exertion and repetitive hand movements (n = 535). (TIF) [file pone.0329324.s006.tif]

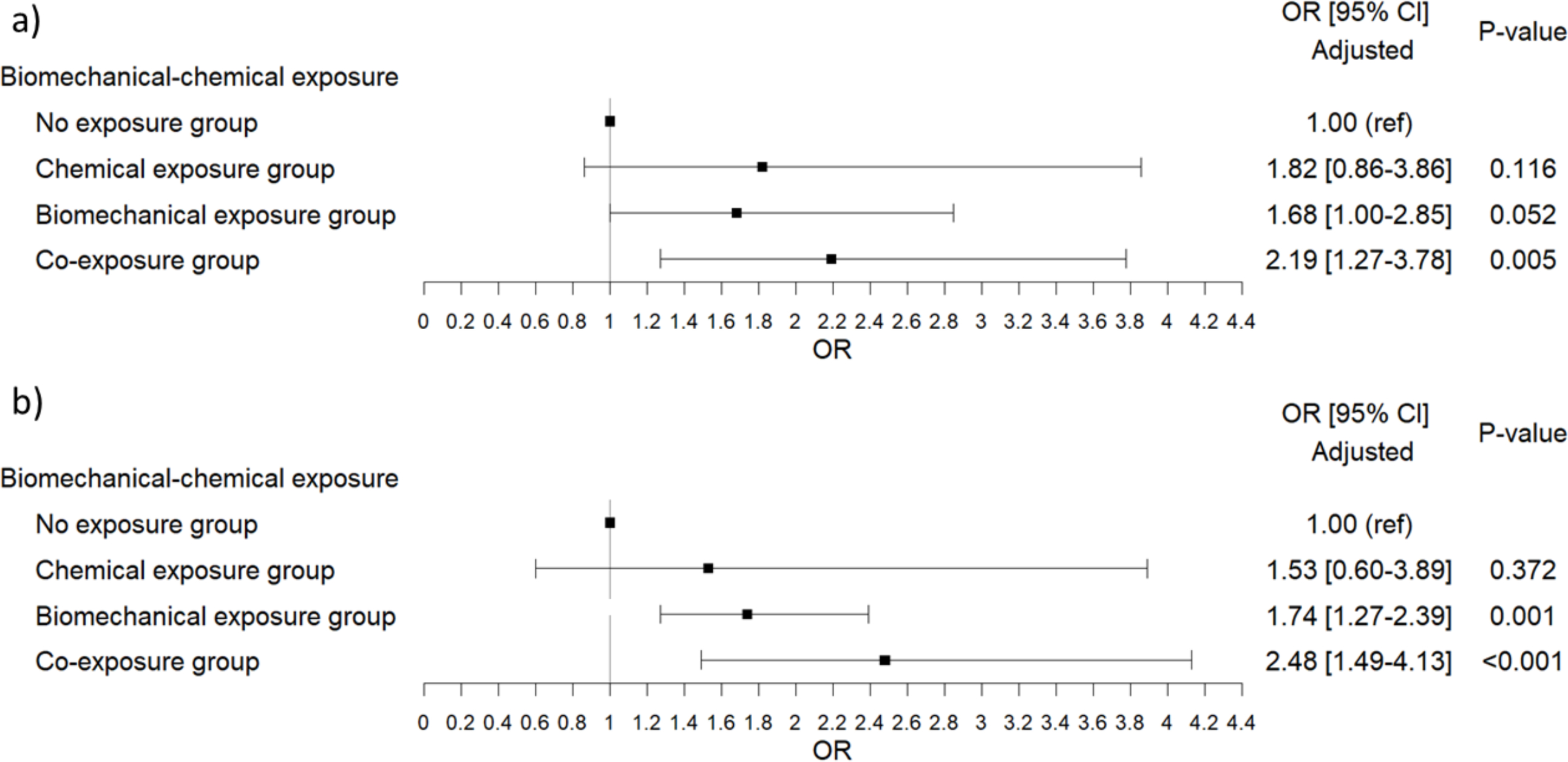

Supplement: S8 Fig — (TIF) [file pone.0329324.s008.tif]

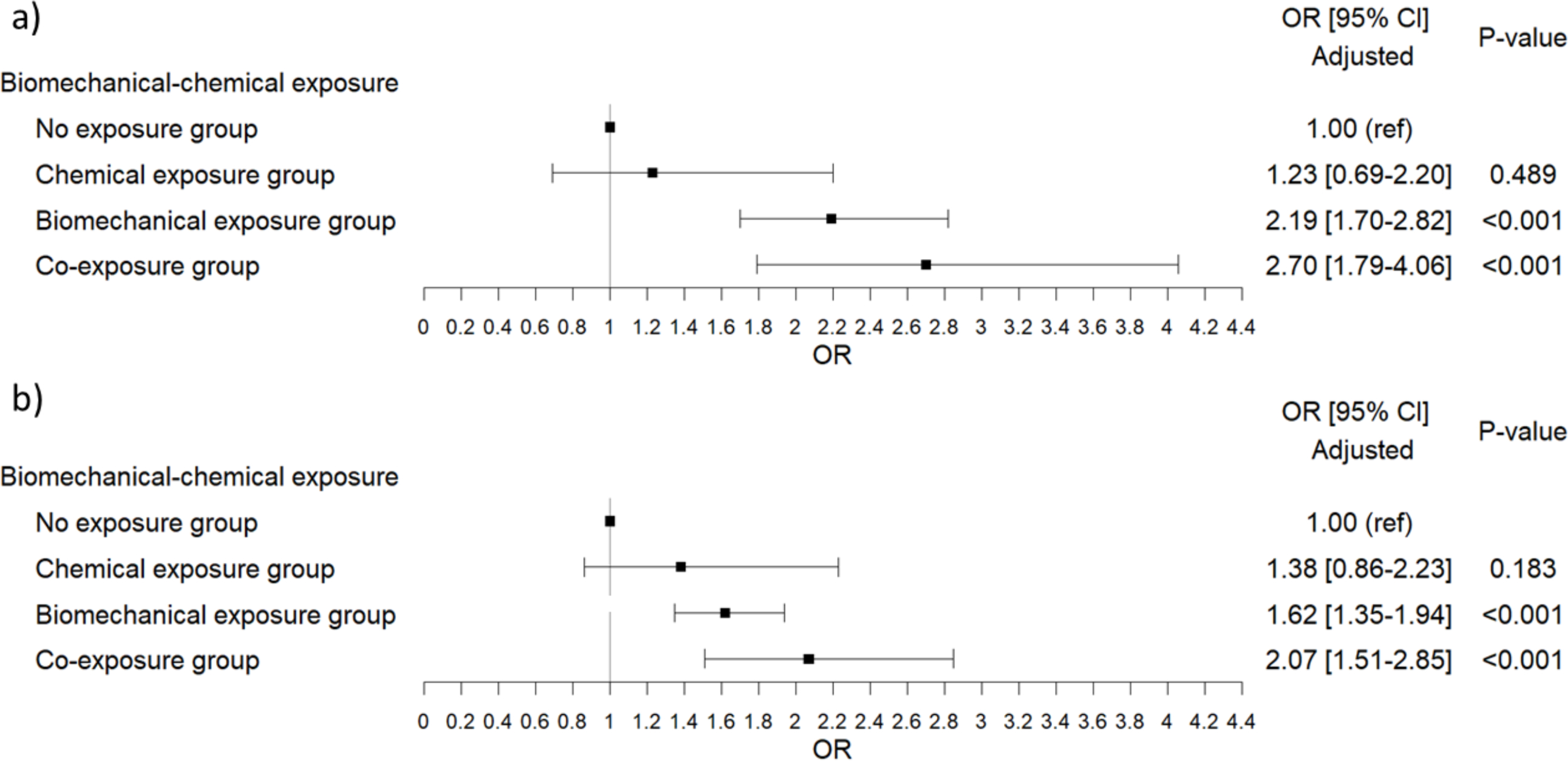

Supplement: S9 Fig — Thresholds: > 2 hours/day for repetitive movements and medium and high exposure to chemical exposure, according to cumulative exposure index (vs no and low exposure). (TIF) [file pone.0329324.s009.tif]
