# Supplementary material for: Carpal tunnel syndrome and occupational co-exposure to biomechanical factors and neurotoxic chemicals using job-exposure matrices and self-reported exposure: Findings from the Constances cohort
Source: PLoS One. 2025 Sep 15;20(9):e0329324. doi: 10.1371/journal.pone.0329324 (PMC12435683; doi:10.1371/journal.pone.0329324)
Supplement: S4 Table — (DOCX) [file pone.0329324.s004.docx]

**S4 Table. Univariate risk models for CTS in men and women**

|  | **Men** | | | **Women** | | |
| --- | --- | --- | --- | --- | --- | --- |
| **AQ** | **Crude OR** | **95% CI** | **p-value** | **Crude OR** | **95% CI** | **p-value** |
| **Biomechanical-chemical exposure (occupational chemical exposure assessed by job-exposure matrices)** |  |  | <0.001 |  |  | <0.001 |
| No exposure | 1 |  |  | 1 |  |  |
| Chemical exposure only | 1.42 | [0.92-2.20] |  | 1.51 | [1.07-2.14] |  |
| Biomechanical exposure only | 2.15 | [1.66-2.80] |  | 1.57 | [1.30-1.90] |  |
| Co-exposure | 2.71 | [1.91-3.84] |  | 2.28 | [1.76-2.96] |  |
| **Biomechanical-chemical exposure (Occupational chemical exposure assessed by self-reported)** |  |  | <0.001 |  |  | <0.001 |
| No exposure | 1 |  |  | 1 |  |  |
| Chemical exposure only | 1.54 | [1.05-2.25] |  | 1.29 | [0.83-1.99] |  |
| Biomechanical exposure only | 2.06 | [1.61-2.63] |  | 1.64 | [1.38-1.95] |  |
| Co-exposure | 3.30 | [2.47-4.39] |  | 2.67 | [1.93-3.69] |  |
