# Supplementary material for: Carpal tunnel syndrome and occupational co-exposure to biomechanical factors and neurotoxic chemicals using job-exposure matrices and self-reported exposure: Findings from the Constances cohort
Source: PLoS One. 2025 Sep 15;20(9):e0329324. doi: 10.1371/journal.pone.0329324 (PMC12435683; doi:10.1371/journal.pone.0329324)
Supplement: Attachment — Submitted filename: PlosOne_Responses.docx [file pone.0329324.s011.docx]

Journal requirements:

When submitting your revision, we need you to address these additional requirements.

1. Please ensure that your manuscript meets PLOS ONE's style requirements, including those for file naming. The PLOS ONE style templates can be found at

<https://journals.plos.org/plosone/s/file?id=wjVg/PLOSOne_formatting_sample_main_body.pdf> and

<https://journals.plos.org/plosone/s/file?id=ba62/PLOSOne_formatting_sample_title_authors_affiliations.pdf>.

We have modified the manuscript according to the recommendations.

2. We note that the grant information you provided in the ‘Funding Information’ and ‘Financial Disclosure’ sections do not match.

 When you resubmit, please ensure that you provide the correct grant numbers for the awards you received for your study in the ‘Funding Information’ section.

We have modified the manuscript according to the “Funding information”.

 3. Thank you for uploading your study's underlying data set. Unfortunately, the repository you have noted in your Data Availability statement does not qualify as an acceptable data repository according to PLOS's standards.

At this time, please upload the minimal data set necessary to replicate your study's findings to a stable, public repository (such as figshare or Dryad) and provide us with the relevant URLs, DOIs, or accession numbers that may be used to access these data. For a list of recommended repositories and additional information on PLOS standards for data deposition, please see <https://journals.plos.org/plosone/s/recommended-repositories>.

A data Availability Statement has been added: *“The data underlying this article were provided by the CONSTANCES cohort under permission and are not publicly available, to ensure the confidentiality of study participants. However, the CONSTANCES cohort is “an open epidemiological laboratory” and access to study protocols and data is available on request (*[*https://www.constances.fr/en/scientific-area/access-to-constances-2/*](https://www.constances.fr/en/scientific-area/access-to-constances-2/)*)”*

We would like to thank the editorial office for their comments. We have carefully reviewed the article to respond to the reviewers' comments. Please find below a detailed response to each of their comments.

Review Comments to the Author

**Reviewer #1:** This manuscript addresses an important and timely topic in occupational health, focusing on the relationship between biomechanical and chemical co-exposures and the development of carpal tunnel syndrome (CTS). The study leverages the CONSTANCES cohort, a unique and robust dataset that integrates comprehensive occupational exposure histories, detailed self-reported information, and job-exposure matrices (JEM). This combination of data sources provides a rare opportunity to explore the complex interactions between multiple workplace exposures and their cumulative effects on workers' health.

What sets this research apart is its focus on the co-exposure paradigm—an underexplored yet critical area in occupational epidemiology. While prior studies have often isolated biomechanical and chemical risks, this work seeks to understand their combined impact, offering a more holistic view of workplace hazards. The findings have the potential to inform targeted interventions and preventive strategies, thereby improving worker health and safety.

By addressing key knowledge gaps and utilizing an innovative methodological approach, this research contributes significantly to the broader understanding of multi-exposure scenarios and their implications for occupational health outcomes. However, as with any study, there are areas for refinement to enhance the clarity, rigor, and practical relevance of the findings. This review highlights both the strengths and the areas needing improvement, providing constructive feedback to further strengthen the manuscript.

We thank Reviewer #1 for his feedback on our manuscript. We greatly appreciate the time and effort he has put into his review. Our specific responses are provided below.

**Conceptual and Theoretical Framework**

Justification of Hypotheses:The hypothesis that biomechanical and neurotoxic chemical exposures synergistically increase the risk of carpal tunnel syndrome (CTS) needs stronger theoretical backing. The concept of the 'double-crush syndrome' is mentioned but not thoroughly supported with detailed mechanistic studies or references. Recommend including recent studies or reviews on neurotoxicity and its impact on peripheral nerve susceptibility to mechanical compression.

According to your remark, we have added references on the association between the association between CTS and chemical exposure and we have detailed the justification of the hypotheses in the Introduction section.

*“To the best of our knowledge, the impact of chemical exposure on the risk of CTS has rarely been studied despite the potential neurotoxicity of some chemicals [15,26–31]. Ophir et al. [29] reported an increased risk of CTS-like symptoms following subclinical sensory polyneuropathy (affecting mainly the median and sural nerves) in workers exposed to prolonged low-level exposure to organophosphate in rural communities in Israel. However, a case-control study of CTS conducted in the general population of Wisconsin failed to report an association between CTS and chemical exposure after adjustment for the known risk factors [26]*

*[…]*

*Systemic peripheral neuropathies (e.g., diabetic polyneuropathy) are known to cause subtle nerve damage, thus rendering the median nerve more vulnerable to compression within the carpal tunnel and against the volar ligament in the case of overexposure to biomechanical stressors [32,33]. Following the same reasoning, exposure to chemicals in the workplace could also cause subclinical changes in the median nerve [29] - producing a form of double crush syndrome that could weaken the median nerve [34]. This could potentiate mechanical stress on the nerve in the event of co-exposure with biomechanical stress factors that increase intra-tunnel pressure. Subclinical changes in the central nervous system (CNS) can also occur in workers exposed to chemicals [35,36] and lead to impaired sensorimotor control of force production and finger dexterity, thus generating greater mechanical stress on the median nerve.”*

Integration of Neurotoxic Chemical Evidence:The paper lacks detailed discussion of the specific neurotoxic pathways of chlorinated solvents and formaldehyde and their relevance to CTS development. Expand on known mechanisms (e.g., inflammation, reactive oxygen species overproduction) and provide references to strengthen the link between chemical exposure and nerve damage.

According to your remark, we have developed the discussion section:

*“Two putative mechanisms, peripheral and central, may be involved in the combined effects of neurotoxic chemical and biomechanical exposures on the risk of CTS. First, systemic peripheral nerve damage (peripheral mechanism), secondary to neurotoxic exposure, could induce diffuse nerve microdamage making the median nerve more sensitive to compression in the carpal tunnel secondary to biomechanical stresses (concept of “double crush syndrome” induced by chemicals) [32–34]. Second, central nervous lesions of neurotoxic origin lead to functional and/or structural alterations of the CNS [19,22,25,35,36,50,59] (Central mechanism), in particular in the sensorimotor zones likely to increase the effects of CTS by (i) impairing sensorimotor coordination and disrupting the biomechanical efficiency of professional movements, and (ii) accentuating the decrease in dexterity induced by anomalies in haptic sensory feedback during CTS [60–62]. These mechanisms could lead to an increase in the biomechanical stresses applied to the wrist, and thus create a vicious circle likely to accentuate the risk of CTS in co-exposed workers.”*

**Methodology**

Self-Reported Bias:Reliance on self-reported CTS and chemical exposure introduces recall and reporting bias, potentially overestimating associations. Suggest validating self-reports with clinical data, such as medical records or surgery databases, and conducting sensitivity analyses to estimate the impact of misclassification. Further describing any methods employed (if any) to minimize these biases would be helpful.

We agree with your remark that self-reported CTS and occupational exposure are problematic. Given the large size of the cohort (over 200,000 adults), diagnostic by physicians was not possible. We have access to French social security database, and a future analysis will be to study associations between surgical CTS and occupational co-exposure. However, there are other treatments for CTS (orthosis, infiltration, etc.) that are difficult to identify in the French social security database, and considering only CTS that have been operated would not allow to identify these “lighter” cases. So, it seems appropriate to also study the association between self-reported CTS and co-exposures. The discussion section has been modified as follows:

*“CTS was self-reported and some participants may have reported CTS without clinical diagnosis. The definition used lacks specificity. However, due to the large size of the cohort, diagnostic by physicians was not possible. Palmer et al. concluded that complex upper limb disorder case definitions (e.g. involving physical signs, more specific symptom patterns, and investigations) yield similar associations with occupational risk factors to those using simpler definitions [65].”*

*“Access to French administrative health care database will enable us to study associations between surgically treated CTS and occupational co-exposures in a future study.”*

Concerning the chemical exposure, we used two methods: JEM and self-report to study associations, and both methods give similar results.

Limited Chemical Scope:The exclusion of other known neurotoxic chemicals (e.g., heavy metals, polycyclic aromatic hydrocarbons) is a significant limitation. Justify this choice in the methods text or explicitly acknowledge it as a limitation in the discussion section.

We agree that selecting chemicals based on the availability of JEM is a limitation. The methods section has been modified as follows:

*“Chemicals studied were selected according to their potential neurotoxicity [17,40–44] and their availability in JEMs [45] and self-administered questionnaires. At the time the analyses were carried out, only the JEMs for chlorinated solvents and formaldehyde were available in the same nomenclatures as the Constances job codes. Chlorinated solvents are known as neurotoxicants [22,44,46–48]. The neurotoxicity of formaldehyde may involve several pathways, including inflammation or overproduction of intracellular reactive oxygen species (ROS) [40,41].”*

The following sentence has been added in the discussion section:

*“In addition, some known neurotoxic chemicals, such as lead, mercury, n-hexane, were not available in JEM. JEMs developed by Santé publique France for petroleum and oxygenated solvents will soon be updated with the same nomenclatures of jobs as Constances, and analyses taking these solvents into account could be carried out in the future.”*

Gender Stratification Rationale:Gender differences in exposure and CTS risk are presented but not deeply analyzed. Explore possible reasons for these differences, such as occupational roles, task-specific exposures, or physiological factors.

The analyses were stratified by gender to take account of the difference in CTS incidence between men and women, and also of the difference in occupational exposures, in line with the recommendations for the study of musculoskeletal disorders. We agree with your remark and we have developed the results and discussion sections as follows:

*“Women were more exposed to biomechanical risk factors than men (32.6% vs. 28.0% respectively, p<0.001). In detail, women reported more high physical demand (21.1% vs. 19.6% respectively, p<0.001) repetitive movements (11.3% vs. 8.2%, p<0.001) and repetitive pinching (4.5% vs. 3.6%, p<0.001), while men were more exposed to awkward wrist postures (7.4% vs. 6.4%, p<0.001) and hand-transmitted vibrations (3.4% vs. 0.9%, p<0.001).”*

*“Analyses were stratified according to gender following recommendations for the study of musculoskeletal disorders [54,55]. The differences in exposure frequency between men and women could be explained by the fact that women and men do not occupy the same jobs. In addition, even within the same occupation, several studies have shown that exposure differs between men and women due to the gendered distribution of tasks, missions and workspaces [63].”*

Potential Confounders:Effort-reward imbalance and alcohol consumption are included as covariates, but other factors (e.g., psychosocial stress, exposure to additional workplace hazards) could confound the results. Consider conducting additional analyses or cite literature supporting the adequacy of the chosen covariates or providing more detailed rationale why they were not considered.

According to your comment and that of the Reviewer #2, we have developed the discussion section:

*“Given the large size of the cohort (over 200,000 adults) and the global nature of the cohort, questions deemed relevant to the study of CTS could not be asked, such as ergonomic measures in the workplace and the wearing of protective equipment. We chose covariates based on the literature [13,51–53], and opted to not include tobacco consumption [51]. Physical activity would have also been relevant. However, the physical activity practiced was not indicated in the questionnaire, and some sports, such as tennis or golf have an impact on CTS, while others have no impact. Endocrine disorders such as hyperthyroidism and hypothyroidism were not available in the questionnaires at baseline. In addition, there is no validated algorithm for identifying these disorders in French administrative health care database. Consequently, these disorders could not be used as covariates.”*

Statistical Model Details:The methodology section does not adequately explain key statistical choices, such as the selection of logistic regression, handling of interaction terms, and treatment of missing data. Include a rationale for these decisions and clarify how missing data were addressed (e.g., multiple imputation or exclusion). Additionally, assessment of potential confounders and the decision process for those should be described further.

According to you remark, more details about the statistical analysis have been added. In addition, multiple imputation was implemented to treat missing data.

*“These covariates were chosen because they were known to be risk factors for CTS [13,51–53].*

*[…]*

*Multiple imputation was performed to handle missing data for covariates (BMI, alcohol use disorders, ERI and diabetes mellitus and/or rheumatoid arthritis) and occupational category with twelve imputed data sets. […] Interactions between co-exposure and age were tested.”*

Threshold Definitions for Exposure:The thresholds for biomechanical exposure (e.g., repetitive movements for >4 hours/day) and chemical exposure categories appear arbitrary. Consider conducting and reporting sensitivity analyses to evaluate whether changing these thresholds affects results.

We have added sensitivity analyses by modifying the thresholds for repetitive movements (>2 hours/day) and for chemical exposure according to cumulative exposure index (medium and high exposure vs no and low exposure), see Methods, Results and S8 Fig.

**Discussion and Conclusion**

Causality Limitations:The results are presented as though they imply causation, despite the inherent limitations of observational studies. Emphasize the associative nature of the findings and discuss potential reverse causality (e.g., pre-existing conditions influencing exposure reports).

According to your remark, we have reformulated the abstract and discussion. We also discuss of potential reverse causality as follow:

*“The measurement of self-reported occupational exposure is a limitation due to potential reverse causality, i.e. subjects with pain tend to declare more expositions. To limit this bias, we have excluded subjects reporting chronic wrist pain and/or CTS at baseline.”*

Overemphasis on Findings:The conclusion overstates the implications of the findings by implying a causal relationship. Adjust language to reflect the observational design and highlight areas where further research is needed to confirm causality.

The conclusion section has been modified as follows:

*“In conclusion, this large prospective study showed an association between incident CTS and co-exposure to both occupational biomechanical risk factors and chemicals, using JEM and self-report methods. These results are consistent with those of our previous studies in the same cohort with self-reported chemical exposure [15], and in a cohort of French farmers, although, we lack mechanistic evidence, provide further support for the potential effects of chemical exposure on the risk of CTS in workers exposed to biomechanical risk factors. Access to French administrative health care database will enable us to study associations between surgically treated CTS and occupational co-exposures in a future study. It would also be relevant to study other chemical exposures (petroleum and oxygenated solvents), as well as biomechanical exposures by considering a more objective measure such as JEM.”*

Study Population Representation:The exclusion of self-employed workers, farmers, and other groups limits the generalizability of the findings. Address this limitation in the discussion and explore the potential impact on results.

According to your remark, we have developed the discussion section:

*“Despite its size, the study sample does not represent the whole structure of occupations in France, as self-employed workers and farmers are not included in the cohort. There are two cohorts set up by Santé publique France (Coset-MSA and Coset-Indépendants) that would allow to study these specific populations [64].”*

Future Research Suggestions provide specific and actionable recommendations for future studies, such as using clinical CTS diagnoses, exploring additional chemical exposures, or utilizing objective biomechanical exposure assessments.

The discussion section has been modified:

*“Access to French administrative health care database will enable us to study associations between surgically treated CTS and occupational co-exposures in a future study. It would also be relevant to study other chemical exposures (petroleum and oxygenated solvents), as well as biomechanical exposures by considering a more objective measure such as JEM.”*

**Ethical and Practical Considerations**

Ethical Concerns in Data Use:The data accessibility restrictions, while necessary for participant confidentiality, hinder replication efforts. Include clear instructions for researchers on how to obtain the necessary permissions and datasets.

A data Availability Statement has been added: *“The data underlying this article were provided by the CONSTANCES cohort under permission and are not publicly available, to ensure the confidentiality of study participants. However, the CONSTANCES cohort is “an open epidemiological laboratory” and access to study protocols and data is available on request (https://www.constances.fr/en/scientific-area/access-to-constances-2/)*

**Typos and Grammar**

Abstract:

- Improper sentence structure: 'cohort that included, between 2012 and 2019, a randomly selected sample of adults.'

To improve clarity, we have revised the sentence to: *“A randomly selected sample of adults were included between 2012 and 2018 in the French cohort CONSTANCES.”*

- Awkward phrasing: 'Lifetime occupational exposure to chemicals was assessed using both methods: JEM and a self-administered questionnaire completed at inclusion.'

To improve clarity, we have revised the sentence to: *“Lifetime occupational exposure to chemicals was assessed using two different methods: with JEMs and with a self-administered questionnaire completed at inclusion.”*

Methods:

- Repetitive statement: 'All volunteers signed a written consent form for their participation in CONSTANCES.'

We are sorry, we did not see where this statement was repeated.

Results:

- Inconsistent spacing in statistical reporting: 'OR = 3.07 [2.28-4.08].'

Spaces have been modified.

Discussion:

- Redundant sentence: 'This finding confirms previous results in the same cohort.'

We have revised the sentences.

**Reviewer #2:** General Comments

The study evaluates the association between occupational co-exposure to biomechanical risk factors and potentially neurotoxic chemicals with carpal tunnel syndrome (CTS) in a cohort of French workers. It is relevant because it addresses one of the most common entrapment neuropathies in workers exposed to biomechanical and chemical factors. Additionally, the use of a large cohort significantly enhances the consistency of the findings and allows for the comparison of different approaches to estimate chemical exposure in the workplace. However, the self-reported diagnosis of CTS may introduce bias. Furthermore, the study does not analyze the intensity or duration of exposure, and there are potential unconsidered confounding factors. Additionally, the data analysis does not specify whether it was adjusted for the type of random sampling used.

We thank Reviewer #2 for his feedback on our manuscript. We greatly appreciate the time and effort he has put into his review. Our specific responses are provided below.

Regarding the writing style, there are spelling errors in all sections of the manuscript that need to be corrected.

According to your remark, the manuscript was proofread by one of the English-speaking co-authors.

**Introduction**

The introduction presents the problem with appropriate wording and is supported by scientific literature from the past 15 years. No major observations were found, except for some spelling errors.

We would like to thank Referee #2 for his/her comments.

**Methods**

The self-reported outcome is the primary limitation of this study. The authors should justify why they chose this approach.

We agree with your remark that self-reported CTS and occupational exposure are problematic. Given the large size of the cohort (over 200,000 adults), diagnostic by physicians was not possible. We have access to French administrative health care database, and a future analysis will be to study associations between surgical CTS and occupational co-exposure. However, there are other treatments for CTS (orthosis, infiltration, etc.) that are difficult to identify in the French social security database, and considering only CTS that have been operated would not allow to identify these cases. So, it seems appropriate to also study the association between self-reported CTS and co-exposures. The discussion section has been modified as follows:

*“CTS was self-reported and some participants may have reported CTS without clinical diagnosis. The definition used lacks specificity. However, due to the large size of the cohort, diagnostic by physicians was not possible. Palmer et al. concluded that complex upper limb disorder case definitions (e.g. involving physical signs, more specific symptom patterns, and investigations) yield similar associations with occupational risk factors to those using simpler definitions [65].”*

*“Access to French administrative health care database will enable us to study associations between surgically treated CTS and occupational co-exposures in a future study.”*

What was the rationale for assessing the outcome at 12 months? While CTS can develop within months or a few years (depending on the high biomechanical demand), most workers develop it over a period exceeding five years. The follow-up period needs to be justified, and if relevant, considered as a potential limitation.

We agree with your remark. However, we are limited to analyze the data available in the first follow-up questionnaires in order to have enough subjects. Each follow-up questionnaire asks about the existence of health problems in the past year (around 40), including CTS.

Regarding covariates, important confounders were not included, such as: Duration of exposure to biomechanical or chemical agents, interventions to reduce CTS risk, such as ergonomic measures, active breaks, or protective equipment, physical activity outside the workplace, smoking habits, and individual factors, such as endocrine disorders or the use of medications affecting nerve function.

These variables should be explicitly detailed in the limitations section.

According to your comment and that of the Reviewer #1, we have developed the discussion section:

*“Given the large size of the cohort (over 200,000 adults) and the global nature of the cohort, questions deemed relevant to the study of CTS could not be asked, such as ergonomic measures in the workplace and the wearing of protective equipment. We chose covariates based on the literature [13,51–53], and opted to not include tobacco consumption [51]. Physical activity would have also been relevant. However, the physical activity practiced was not indicated in the questionnaire, and some sports, such as tennis or golf have an impact on CTS, while others have no impact. Endocrine disorders such as hyperthyroidism and hypothyroidism were not available in the questionnaires at baseline. In addition, there is no validated algorithm for identifying these disorders in French administrative health care database. Consequently, these disorders could not be used as covariates.”*

Concerning the duration of exposure to biomechanical factors, the median duration of current job was 8 years (IQR 4-16) for men and 9 years (IQR 4-16) for women. The duration of exposure in years to biomechanical factors was not asked in the questionnaire.

The statistical analysis does not indicate whether the calculations were adjusted for the random sampling method declared in the study. If not accounted for, this could significantly impact the results. Additionally, the authors should clarify whether they employed any strategies to handle missing data.

The statistical analysis was not performed using weighted data. Indeed, the weights were calculated by year of inclusion by the research team in charge of the CONSTANCES cohort, i.e. the 2013 weighting extrapolates the results obtained for the CONSTANCES target population in 2013, and the 2014 weighting extrapolates the results obtained for the CONSTANCES target population in 2014. It is not possible to combine the years of inclusion. In order to have a sufficient number of CTS cases, we have chosen to study workers included between 2012 and 2018.

According to you remark, more details about the statistical analysis have been added. In addition, multiple imputation was implemented to treat missing data.

*“Multiple imputation was performed to handle missing data for covariates (BMI, alcohol use disorders, ERI and diabetes mellitus and/or rheumatoid arthritis) and occupational category with twelve imputed data sets.”*

The study mentions a sex-stratified analysis, but no justification is provided. Stratification by type of occupational activity could provide more specific risk measures. The authors should evaluate this possibility.

We agree with your remark and we have developed reasons for these differences. The results and discussion sections have been modified as follows:

*“Analyses were stratified according to gender following recommendations for the study of musculoskeletal disorders [54,55]. The differences in exposure frequency between men and women could be explained by the fact that women and men do not occupy the same jobs. In addition, even within the same occupation, several studies have shown that exposure differs between men and women due to the gendered distribution of tasks, missions and workspaces [63].”*

A sensitivity analysis was performed for a subpopulation restricted to low-grade white-collar workers and blue-collar workers. The associations were globally similar (see S6 Fig and S7 Fig).

The authors used logistic regression models adjusted for confounders; however, given the low frequency of the outcome (CTS), an alternative approach would be to use Firth logistic regression, which corrects the overestimation of odds ratios (ORs).

In line with your comment, we have tested Firth logistic regression. The results are quite similar and we have kept the results of the logistic regression.

| **JEM** | OR | 95% CI | |
| --- | --- | --- | --- |
| **Men** |  |  |  |
| No exposure group | 1 |  |  |
| Chemical exposure group | 1.11 | 0.65 | 1.78 |
| Biomechanical exposure group | 2.03 | 1.53 | 2.68 |
| Co-exposure group | 2.39 | 1.61 | 3.46 |
| **Women** |  |  |  |
| No exposure group | 1 |  |  |
| Chemical exposure group | 1.52 | 1.03 | 2.17 |
| Biomechanical exposure group | 1.48 | 1.20 | 1.83 |
| Co-exposure group | 2.09 | 1.55 | 2.77 |
|  |  |  |  |
|  |  |  |  |
| **Self-administered questionnaire** |  |  |  |
|  | OR | 95% CI | |
| **Men** |  |  |  |
| No exposure group | 1 |  |  |
| Chemical exposure group | 1.19 | 0.75 | 1.79 |
| Biomechanical exposure group | 1.85 | 1.41 | 2.40 |
| Co-exposure group | 3.12 | 2.29 | 4.21 |
| **Women** |  |  |  |
| No exposure group | 1 |  |  |
| Chemical exposure group | 1.42 | 0.87 | 2.18 |
| Biomechanical exposure group | 1.53 | 1.26 | 1.85 |
| Co-exposure group | 2.47 | 1.70 | 3.48 |

**Results**

The authors should indicate whether any workers were excluded during the follow-up period and provide reasons for their exclusion, as Figure S1 does not mention this.

According to your remark, we have modified the flowchart and restricted the population to those included between 2012 and 2018, as few of those included between 2019 and 2020 had a follow-up questionnaire. The participation rate in follow-up was 76%.

In Table 2, some data are in French, and the translation should be completed. Additionally, the p-values should include superscripts to specify the statistical test used.

We thank Referee #2 for his detailed feedback on our manuscript, the Tables 1 and 2 have been modified.

**Discussion**

The authors should explain how exposure misclassification could lead to an underestimation or overestimation of individual exposure and propose strategies to overcome this limitation.

In using self-administered questionnaire, workers might over or underestimate occupational exposures, leading to exposure misclassification. However, observation or direct measurement are expensive and difficult to apply to large cohorts. The assessment of occupational factors was asked for a typical workday of the preceding 12-month period. Moreover, awkward postures were presented in picture form to facilitate workers’ understanding and increase the validity of posture self-assessment. The discussion section has been modified. The discussion section has been modified.

Regarding the outcome (CTS diagnosis), the authors should consider discussing more objective assessment alternatives, such as: validated clinical algorithms, electromyographic testing, and medical records of carpal tunnel release surgery.

Incorporating these aspects would strengthen the validity of the findings and minimize potential biases in CTS classification.

We agree with your remark that self-reported CTS is problematic. Given the large size of the cohort (over 200,000 adults), diagnostic by physicians was not possible. We have access to French social security databases, and a future analysis is to study associations between surgical CTS and occupational co-exposure. However, not all people with CTS undergo surgery (around a third), and it seems appropriate to also study the association between self-reported CTS and co-exposures. The discussion section has been modified as follows:

*“Access to French administrative health care database will enable us to study associations between surgically treated CTS and occupational co-exposures in a future study.”*
