# Supplementary material for: Carpal tunnel syndrome and occupational co-exposure to biomechanical factors and neurotoxic chemicals using job-exposure matrices and self-reported exposure: Findings from the Constances cohort
Source: PLoS One. 2025 Sep 15;20(9):e0329324. doi: 10.1371/journal.pone.0329324 (PMC12435683; doi:10.1371/journal.pone.0329324)
Supplement: Attachment — Submitted filename: Response to Reviewers.docx [file pone.0329324.s012.docx]

**Editor**

Thank you for submitting your manuscript to PLOS ONE. After careful consideration, we feel that it has merit but does not fully meet PLOS ONE’s publication criteria as it currently stands. Therefore, we invite you to submit a revised version of the manuscript that addresses the points raised during the review process.

More in details, carefully address the points raised by the reviewer #3 who expressed fundamental concerns about the suitability of the CONSTANCES dataset for investigating carpal tunnel syndrome (CTS). They argue that CTS is not only highly prevalent but also widely recognized—often inaccurately—by the general public. This widespread familiarity introduces significant potential for reporting and recall bias that I strongly recommend to discuss in the discussion section also by providing hypothetical estimations of how they might have affected the presented results. In particular, the Authors should frankly discuss the potential distortion in the data (quantitevely) in the context of small observed differences (1–4%) in CTS incidence between exposure groups (they might not hold, and this must be addressed). Another important issue refers to the absence of objective diagnostic tools such as nerve conduction studies or ultrasound, that hampers the assessment of the true nature—additive or synergistic—of the relationship between mechanical and chemical exposures and CTS. This point must be also carefully discussed. More broadly,  the Authors must discuss all the limitations of the study, including in the abstract; any unsupported statements of causation must be toned down and the confounding effect should be clearly reported.

We would like to thank you for your comments. We have reviewed the article to respond to your comments and those of reviewers. We have developed the limitations in the discussion paragraph, particularly with regard to the self-reported nature of CTS and the absence of objective diagnostic tools such as nerve conduction studies or ultrasound, and how this might affect the results. We discussed reporting and recall bias.

*“Epidemiological studies include several definitions of CTS [65]. In our study, CTS was reported by participants using a single question (yes/no). We cannot rule out misclassification, as CTS may have been over-reported by workers who believe they have been exposed to occupational factors causing CTS, whether or not their hand symptoms resemble those of CTS. The cohort design did not allow for the use of a symptom algorithm, physical examination or EMG confirmation, which is an important limitation of the study. The working population studied probably did not include the most severe cases of CTS that may have been excluded from the labor market (i.e., the healthy worker effect), which could lead to an underestimate of the prevalence of CTS.*

*Measuring self-reported occupational exposure is an important limitation of the study. Direct observation or measurement is costly and difficult to apply to large cohorts. In addition, the assessment of biomechanical risk factors was requested for a typical working day and was based on the recommendations of the European criteria document for the relatedness of MSDs [38], and postures were presented in pictorial form to facilitate workers' understanding and increase the validity of posture self-assessment. We cannot exclude that biomechanical and chemical exposures were over-reported by workers reporting work-related CTS, which is a potential source of reverse causality. To limit this bias, we have excluded subjects reporting chronic wrist pain and/or CTS at baseline. Nevertheless, there is no evidence to suggest a differential bias depending on whether workers were exposed to biomechanical factors alone or to biomechanical and chemical factors combined. Moreover, the prevalence of self-reported CTS was of the same order of magnitude depending on whether there was biomechanical exposure alone or biomechanical and chemical co-exposure (in analysis with JEM: 2.5% vs 3.1% in men and 3.2% vs 4.5% in women; in analysis with self-report: 2.3% vs 3.6% in men and 3.3% vs 5.2% in women). Although the difference in prevalence was relatively modest, we do not believe that classification bias can explain the results, given the consistency of the results with a subjective assessment and an objective assessment of chemical exposure by JEM.”*

**Review Comments to the Author**

Please use the space provided to explain your answers to the questions above. You may also include additional comments for the author, including concerns about dual publication, research ethics, or publication ethics. (Please upload your review as an attachment if it exceeds 20,000 characters)

**Reviewer #3:** I appear to be coming to this paper as a third reviewer after two very comprehensive reviews by others and a round of revision. The previous reviewers have made many valid minor points about the methodology and presentation, most of which have been adequately dealt with but the elephant in the room here is the self-reported diagnosis of both CTS and some of the measures of occupational exposure. Retrospective analyses of large datasets which have been collected for other purposes is always difficult because they never include all the covariates which you would like to have available in order to answer your particular question but in this case I suspect that the unreliability of the diagnoses is so great that none of the analyses here can really be relied upon.

The problem here is that CTS is not just very common but a disorder which is very widely known to the population at large, who are regularly misinformed by the popular press, social media and ordinary interactions with co-workers and their social circle regarding its symptoms and aetiology. The perception of CTS in the general population, combined with the natural human tendency to seek an explanation for anything that happens to us medically in our previous history presents such potent opportunities for bias in the conduct of this study that I have to say that I would not even have attempted it using this dataset as anything other than a preliminary preparation for a carefully constructed prospective study. “CTS” will be systematically over-reported by patients who believe that they have been exposed to occupational factors that cause CTS regardless of whether their hand symptoms actually bear any resemblance to those of CTS or not, and exposure to mechanical and chemical “causes” of CTS will be equally over-reported by those patients who believe that someone/something must be responsible for their problem resulting in a spurious correlation between CTS and occupational variables. Long exposure to talking to thousands of patients with “CTS” in the clinic would suggest that these biases could easily introduce errors of +/- 20% into the numbers derived from the CONSTANCES cohort data – completely dwarfing the small differences in incidence – in the range of 1-4% - found between the exposure and non-exposure groups in figures 1 and 2. These small differences could easily be a simple result of the effect of popular ‘knowledge’ about CTS on reporting rather than a true difference resulting from causation of CTS by either mechanical or chemical exposure – though I think the existing body of literature does in fact provide evidence for a weak effect of mechanical occupational factors in causing CTS. This problem even extends to diagnoses of CTS made in primary care where there is a marked tendency to overdiagnose the disorder because of GP beliefs about CTS. The JEM data is at least more objective but unfortunately only comprises a small amount of the overall chemical exposure data and even that has some limitations which have already been pointed out by the earlier reviewers.

This fundamental problem also makes it impossible to assess whether, if there is indeed a risk of CTS posed by both mechanical and chemical factors, whether the overall effect is simply summative or synergistic – the topic of much speculation in the discussion section.

I’m sorry to pour cold water on Prof. Roquelaure and team’s endeavours here, as they have done much interesting work on the epidemiology of CTS, but I really think they have not given sufficient thought to the potential problems for this study posed by widespread beliefs about CTS in the working population. I think this study could only realistically be done using objectively determined CTS – which would require NCS or ultrasound data or both. This would miss the small number of cases which are false negative on testing but at least this bias would be small and would apply equitably across occupational exposure groups. In the absence of objective diagnosis of CTS in the CONSTANCES dataset I am afraid I would have concluded on first thinking about this study that this dataset was fundamentally unsuited for this purpose.

We thank Reviewer #3 for his feedback on our manuscript. We greatly appreciate the time and effort he has put into his review. We have tried to review the article to respond to your comments. We have developed the limitations in the discussion paragraph, particularly with regard to the self-reported nature of CTS and the absence of objective diagnostic tools such as nerve conduction studies or ultrasound, and how this might affect the results. We discussed reporting and recall bias.

*“Epidemiological studies include several definitions of CTS [65]. In our study, CTS was reported by participants using a single question (yes/no). We cannot rule out misclassification, as CTS may have been over-reported by workers who believe they have been exposed to occupational factors causing CTS, whether or not their hand symptoms resemble those of CTS. The cohort design did not allow for the use of a symptom algorithm, physical examination or EMG confirmation, which is an important limitation of the study. The working population studied probably did not include the most severe cases of CTS that may have been excluded from the labor market (i.e., the healthy worker effect), which could lead to an underestimate of the prevalence of CTS.*

*Measuring self-reported occupational exposure is an important limitation of the study. Direct observation or measurement is costly and difficult to apply to large cohorts. In addition, the assessment of biomechanical risk factors was requested for a typical working day and was based on the recommendations of the European criteria document for the relatedness of MSDs [38], and postures were presented in pictorial form to facilitate workers' understanding and increase the validity of posture self-assessment. We cannot exclude that biomechanical and chemical exposures were over-reported by workers reporting work-related CTS, which is a potential source of reverse causality. To limit this bias, we have excluded subjects reporting chronic wrist pain and/or CTS at baseline. Nevertheless, there is no evidence to suggest a differential bias depending on whether workers were exposed to biomechanical factors alone or to biomechanical and chemical factors combined. Moreover, the prevalence of self-reported CTS was of the same order of magnitude depending on whether there was biomechanical exposure alone or biomechanical and chemical co-exposure (in analysis with JEM: 2.5% vs 3.1% in men and 3.2% vs 4.5% in women; in analysis with self-report: 2.3% vs 3.6% in men and 3.3% vs 5.2% in women). Although the difference in prevalence was relatively modest, we do not believe that classification bias can explain the results, given the consistency of the results with a subjective assessment and an objective assessment of chemical exposure by JEM.”*

**Reviewer #4:** abstract Clearly signals the dual‑exposure approach (biomechanics + chemicals) and the prospective CONSTANCES cohort.and Gives sex‑stratified headline numbers up‑front. in Introduction Authors now give a fuller rationale for “double‑crush” and cite new mechanistic papers on neurotoxicity

  Methods

• Triangulation: combines a probabilistic Job‑Exposure Matrix (objective, standardised) with self‑reported product use (worker‑level nuance).

• Biomechanical exposures measured through validated CONSTANCES questions based on national ergonomic guidelines.

• Creation of cumulative‑exposure indices (CEI) for chlorinated solvents and formaldehyde introduces quantitative dose information.

 Design & Follow‑up

Participants contributed a single outcome measurement 6‑18 months post‑enrolment. CTS often develops over several years; a one‑year horizon limits temporality and raises reverse‑causation concerns (symptomatic workers may over‑report exposures or join low‑strain jobs).

The authors explain the short window was dictated by available questionnaires ,but do not explore analytical remedies (e.g., landmark analysis, lagged exposure).

CTS is identified by a single yes/no question on self‑reported doctor or self‑perceived CTS

. No symptom algorithm, physical examination, or EMG confirmation is used, inviting non‑differential misclassification that would typically bias estimates toward the null; however differential recall could inflate associations.

Lifetime self‑report asks a single ever/never question for seven broad product groups—susceptible to recall error and left‑censoring of early careers.

Main models are sex‑stratified multivariable logistic regressions

Low outcome frequency (≤2.6 %) raises small‑sample bias; reviewers suggested Firth logistic regression and authors report comparable estimates but retain conventional models

Sampling weights for the CONSTANCES complex design are still not applied; authors state annual weights cannot be pooled, yet robust (sandwich) variance estimators or post‑stratification could have mitigated design effects

Missing data – Multiple imputation (m = 12) is now described, but convergence diagnostics and fraction of missing information are not given

study Uses clear, separate tables for men and women—important because exposure profiles differ markedly by sex.

• Provides both crude and adjusted odds ratios with 95 % CIs, making it easy to gauge confounding impact.

• Forest‑plot figure visually integrates JEM and self‑report estimates side‑by‑side.

Authors now explicitly flag self‑report bias, residual confounding, and limited chemical scope.

While the study retains certain limitations—including a short follow-up window that may limit temporality, reliance on a single self-reported CTS question without clinical confirmation, potential exposure misclassification due to recall error, and the absence of sampling weights in the analysis—the authors have addressed many key methodological concerns in a reasonable and transparent manner. They now provide a clearer biological rationale for the hypothesised double-crush mechanism, apply multiple imputation to handle missing data, and incorporate sex-stratified logistic regression models with additional sensitivity analyses, such as Firth correction and threshold variation, to test the robustness of their findings. Although further refinement—such as the application of robust variance estimators and more detailed validation of the outcome—would strengthen the work, the study’s strengths, including its use of a large, prospective cohort and the integration of both JEM-based and self-reported exposures, make it a valuable and relevant contribution to the literature. On balance, the manuscript is scientifically sound and acceptable for publication.

We thank Reviewer #4 for his feedback on our manuscript. We greatly appreciate the time and effort he has put into his review. We have tried to review the article to respond to your comments. We have developed the limitations in the discussion paragraph, particularly with regard to the self-reported nature of CTS and the absence of objective diagnostic tools such as nerve conduction studies or ultrasound, and how this might affect the results.

The mean and SD of imputed variables were used to check convergence. The plots indicated that convergence had been achieved. Fraction of missing information varies from 0 to 11% depending on the model. A table with crude odds ratios was added in supplementary material.

**Reviewer #5:** This well-conducted, large-scale cross-sectional analysis of the French CONSTANCES cohort (n > 84,000) makes a valuable contribution to occupational health research by examining the joint effects of biomechanical stressors and organic solvent exposure on carpal tunnel syndrome (CTS). The authors employ rigorous methods, including modified Poisson regression and thorough confounder control, and adhere closely to the STROBE guidelines.

Their finding that combined exposure significantly increases the prevalence of self‑reported CTS in both men and women, even after adjusting for a wide range of variables, is both novel and clinically pertinent. The manuscript is lucidly written, and its transparent discussion of limitations—particularly the cross‑sectional design and reliance on self‑report—is commendable.

The authors are invited to consider the following minor suggestions for potential refinement rather than essential revisions.

1. Abstract's Conclusion Clarification: Ensure that readers instantly grasp the context of the results. For example, an additional phrase like "... co‑exposure associated with a higher prevalence of self-reported CTS."

2. Consider a Brief Discussion on Misclassification Bias Direction: In the limitations section, the authors correctly identify the risk of non-differential misclassification bias associated with using self-reported data. They could briefly add that this type of bias typically biases the effect estimate (in this case, the prevalence ratio) towards the null (i.e., towards 1.0). This would strengthen their findings by suggesting that the true association might be even stronger than what they observed.

3. Elaborate on the "Healthy Worker Effect": The authors briefly mention the healthy worker effect. It might be helpful to add one sentence explaining to a broader audience how this could affect their results—specifically, that it could lead to an underestimation of the true prevalence of CTS in the working population, as those who develop severe CTS may have already left the workforce and thus missing from the sample.

We thank Reviewer #5 for his feedback on our manuscript. We greatly appreciate the time and effort he has put into his review. We have reviewed the article to respond to your comments.
